# The DnaA Cycle in *Escherichia coli*: Activation, Function and Inactivation of the Initiator Protein

**DOI:** 10.3389/fmicb.2017.02496

**Published:** 2017-12-21

**Authors:** Tsutomu Katayama, Kazutoshi Kasho, Hironori Kawakami

**Affiliations:** Department of Molecular Biology, Graduate School of Pharmaceutical Sciences, Kyushu University, Fukuoka, Japan

**Keywords:** DnaA, *oriC*, *DARS*, *datA*, chromosome replication

## Abstract

This review summarizes the mechanisms of the initiator protein DnaA in replication initiation and its regulation in *Escherichia coli*. The chromosomal origin (*oriC*) DNA is unwound by the replication initiation complex to allow loading of DnaB helicases and replisome formation. The initiation complex consists of the DnaA protein, DnaA-initiator-associating protein DiaA, integration host factor (IHF), and *oriC*, which contains a duplex-unwinding element (DUE) and a DnaA-oligomerization region (DOR) containing DnaA-binding sites (DnaA boxes) and a single IHF-binding site that induces sharp DNA bending. DiaA binds to DnaA and stimulates DnaA assembly at the DOR. DnaA binds tightly to ATP and ADP. ATP-DnaA constructs functionally different sub-complexes at DOR, and the DUE-proximal DnaA sub-complex contains IHF and promotes DUE unwinding. The first part of this review presents the structures and mechanisms of *oriC*-DnaA complexes involved in the regulation of replication initiation. During the cell cycle, the level of ATP-DnaA level, the active form for initiation, is strictly regulated by multiple systems, resulting in timely replication initiation. After initiation, regulatory inactivation of DnaA (RIDA) intervenes to reduce ATP-DnaA level by hydrolyzing the DnaA-bound ATP to ADP to yield ADP-DnaA, the inactive form. RIDA involves the binding of the DNA polymerase clamp on newly synthesized DNA to the DnaA-inactivator Hda protein. In *datA*-dependent DnaA-ATP hydrolysis (DDAH), binding of IHF at the chromosomal locus *datA*, which contains a cluster of DnaA boxes, results in further hydrolysis of DnaA-bound ATP. SeqA protein inhibits untimely initiation at *oriC* by binding to newly synthesized *oriC* DNA and represses *dnaA* transcription in a cell cycle dependent manner. To reinitiate DNA replication, ADP-DnaA forms oligomers at DnaA-reactivating sequences (*DARS1* and *DARS2*), resulting in the dissociation of ADP and the release of nucleotide-free apo-DnaA, which then binds ATP to regenerate ATP-DnaA. *In vivo, DARS2* plays an important role in this process and its activation is regulated by timely binding of IHF to *DARS2* in the cell cycle. Chromosomal locations of *DARS* sites are optimized for the strict regulation for timely replication initiation. The last part of this review describes how DDAH and DARS regulate DnaA activity.

## Introduction

The genome of *Escherichia coli* consists of a single circular 4.6 Mb chromosome, with a unique replication origin called *oriC*. Replication initiation at *oriC* results in construction of a pair of replisomes, which migrate bi-directionally to replicate the entire chromosome. Replication initiation at *oriC* is regulated to occur only once during each cell cycle, and the timing of initiation is coordinated with the cellular growth rate. Even when cells grow rapidly and the copy number of *oriC* increases to more than two per cell, initiation occurs at sister *oriC* regions simultaneously only once at a specific time during the cell cycle. As such, the time of initiation at *oriC* is regulated and re-initiation during the same cell cycle is strictly repressed (Skarstad and Katayama, 2013; Wolański et al., 2015; Riber et al., 2016).

**FIGURE 1:**
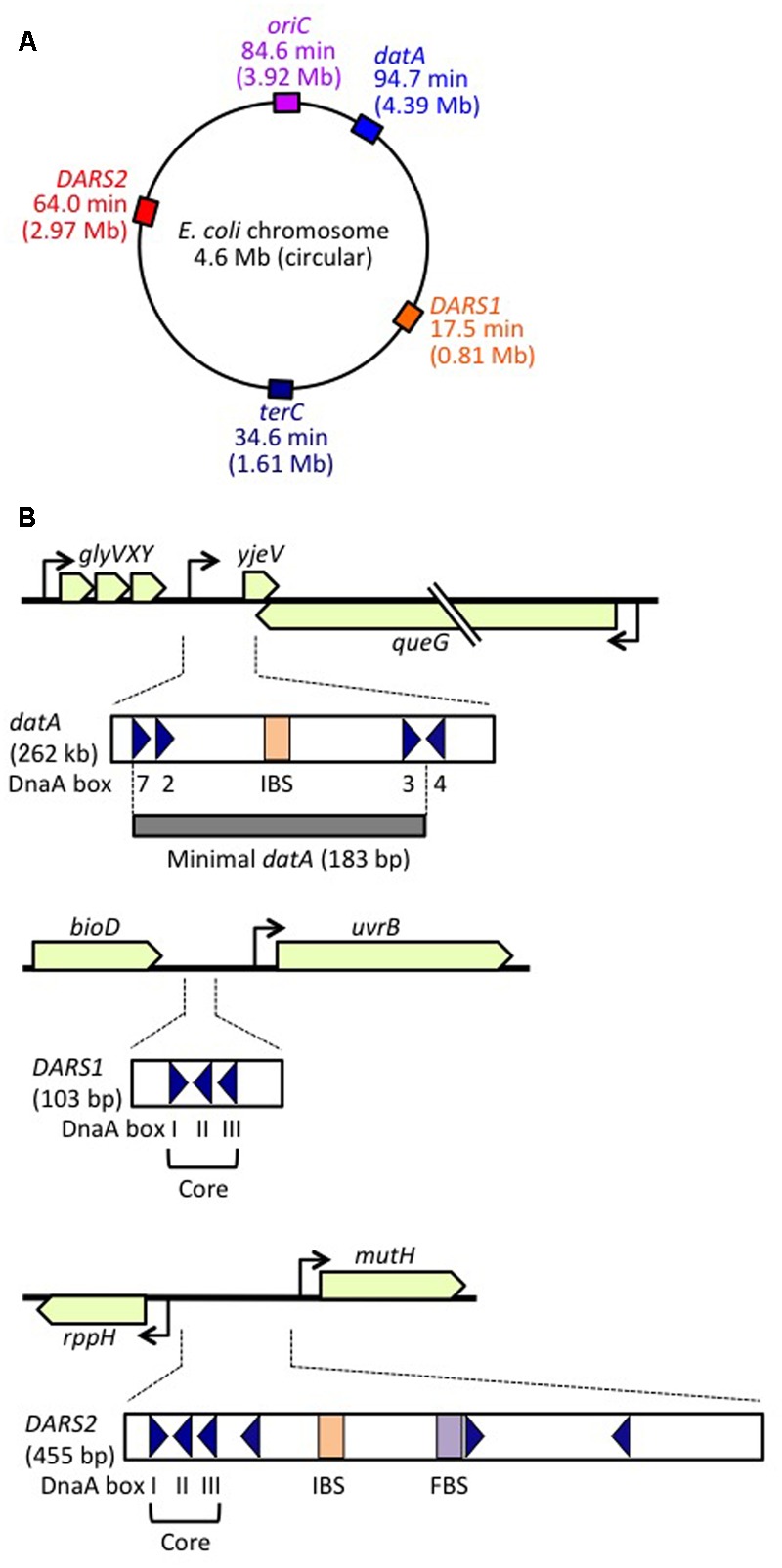
Regulatory DNA elements involved in replication initiation on the *E. coli* genome. **(A)** Schematic representation of the genomic loci of *oriC, datA, DARS1*, and *DARS2* (and *terC*) in the 4.6 Mb circular *E. coli* genome, with positions also indicated on the scale of 0–100 min. **(B)** Structures of *datA, DARS1*, and *DARS2*. Open or gray bars indicate minimal regions. Triangles represent 9 bp DnaA-binding sites (DnaA boxes). Filled squares represent IHF-binding sites (IBS; shown in orange) and a Fis-binding site (FBS; shown in purple). Minimal *datA* consists of DnaA boxes 2, 3, and 7 and a single IBS. DnaA box 4 stimulates DDAH *in vitro*. *DARS1* and *DARS2* both have core regions containing DnaA boxes I–III. *DARS2* also contains additional DnaA boxes and regulatory IBS and FBS.

**FIGURE 2:**
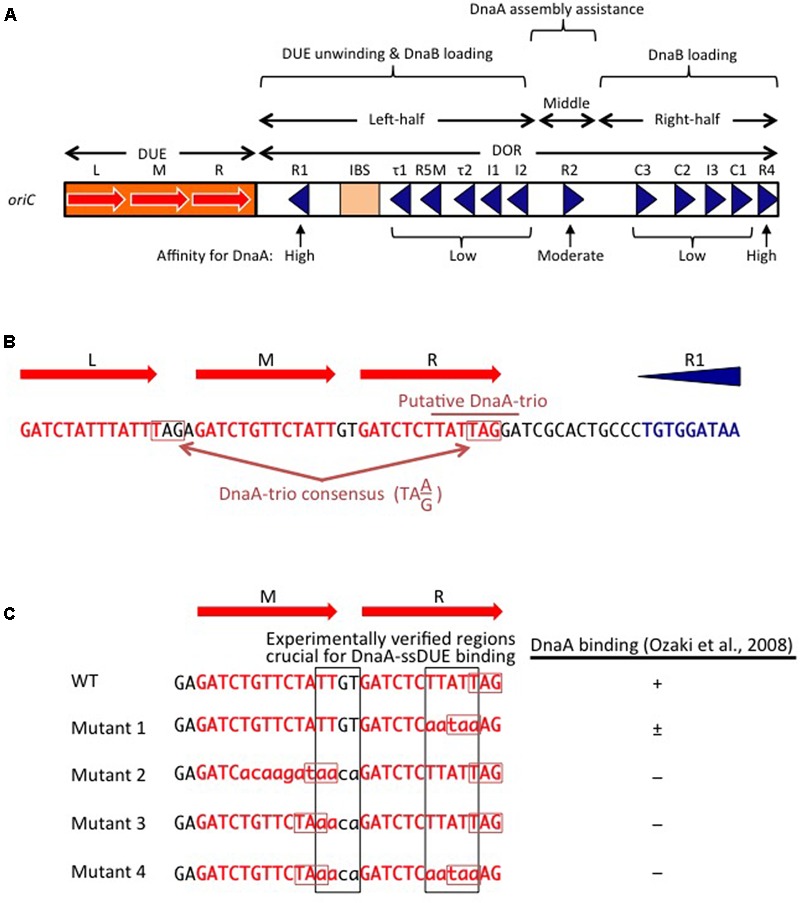
Basic features of *oriC*. **(A)** Structure of *oriC*. Features of the minimal 245 bp *oriC* sequence are shown, including DnaA boxes (triangles), IHF-binding site (IBS; rectangle), and duplex-unwinding element (DUE) AT-rich 13 bp elements (red arrows). Boxes with high, moderate, and low affinity for DnaA are indicated. **(B)** A portion of the *oriC* sequence including the DUE and R1 is shown in detail, with the putative DnaA-trio indicated (Richardson et al., 2016). Perfect DnaA-trio consensus sequences are boxed. **(C)** Effect of mutations on single-stranded DUE (ssDUE) DnaA binding. A portion of the DUE including the M and R 13-mers is indicated in red. Wild-type sequences and mutations are in uppercase and lowercase, respectively. Perfect DnaA-trio consensus sequences are boxed in red. DnaA binding with the indicated ssDUE sequences is summarized (Ozaki et al., 2008). Experimentally verified regions essential for DnaA-ssDUE binding are boxed in black (i.e., TTGT and TTATTT). Note that even if the DnaA-trio consensus is preserved, mutations in the boxed sequences abolish DnaA binding.

The 245 bp minimal *oriC* region has multiple binding sites for the chromosomal replication initiator protein DnaA (DnaA boxes), and a single binding site for the integration host factor (IHF), in addition to an AT-rich duplex-unwinding element (DUE) (**Figures 1A, 2A**; Kaguni, 2011; Leonard and Grimwade, 2015; Wolański et al., 2015; Shimizu et al., 2016). The 9-mer DnaA box consensus sequence is 5′-TTATnCACA-3′. DnaA-initiator-associating protein DiaA is a DnaA-binding protein that stimulates ATP-bound DnaA (ATP-DnaA) assembly on *oriC*, and that is required for timely replication initiation (Ishida et al., 2004; Keyamura et al., 2007). Binding of IHF to DNA causes a sharp (120–180°) bend in the double helix (Swinger and Rice, 2004). A complex consisting of *oriC*, IHF, DiaA, and oligomeric ATP-DnaA is considered to make up the initiation complex in *E. coli* (Keyamura et al., 2007, 2009). This complex unwinds the *oriC* DUE, enabling loading of DnaB helicases onto single-stranded DNA (ssDNA) by specific protein–protein interactions and dissociations, which in turn leads to formation of replisomes (for a review, see Bell and Kaguni, 2013).

Multiple negative and positive regulatory systems target the *oriC*, the *dnaA* gene, and DnaA and work harmoniously to ensure that initiation occurs in a timely manner, in some cases via negative feedback from DNA replication. As for *oriC*, the minimal region contains 11 sites with 5′-GATC-3′ sequences that are specific targets of DNA adenine methylase (Dam) (Waldminghaus and Skarstad, 2009). As GATC is palindromic in duplex DNA, A residues in both strands can be methylated. In newly replicated DNA, only the sites on the parental strand are methylated, whereas those on the daughter strand are unmethylated. The hemimethylated state of *oriC* persists for ∼10 min in cells with a doubling time of 30 min depending on SeqA protein (Lu et al., 1994). SeqA protein has an N-terminal self-oligomerization domain and a C-terminal DNA-binding domain, and binds to the hemimethylated sites, forming self-oligomers. *oriC*–SeqA complexes inhibit binding of DnaA to *oriC*, blocking re-initiation from newly replicated DNA. SeqA sequestration is, therefore, a negative-feedback system coupled to DNA replication (Waldminghaus and Skarstad, 2009; Skarstad and Katayama, 2013). In another negative-feedback system, transcription of *dnaA* is autoregulated by DnaA, and is also repressed by SeqA–Dam-dependent post-replicative regulation (Campbell and Kleckner, 1990; Bogan and Helmstetter, 1997; Speck et al., 1999; Waldminghaus and Skarstad, 2009). These SeqA mechanisms have been well documented elsewhere (Waldminghaus and Skarstad, 2009), and this review will focus instead on regulation of DnaA protein.

*Escherichia coli* cells have at least three regulatory systems for DnaA activity (Skarstad and Katayama, 2013; Riber et al., 2016). In regulatory inactivation of DnaA (RIDA), ATP-DnaA is inactivated in a negative-feedback manner coupled to DNA replication (Katayama et al., 2010). In this system, the clamp subunit of DNA polymerase III holoenzyme has a key role; it remains on the nascent DNA strand after Okazaki-fragment completion, and the clamp–DNA complex binds to the ADP form of DNA regulatory inactivator Hda protein, an ‘ATPases associated with various cellular activities’ (AAA+) protein with an N-terminal clamp-binding site (Katayama et al., 1998; Kato and Katayama, 2001; Su’etsugu et al., 2008; Baxter and Sutton, 2012; Kim et al., 2017). The resultant ADP-Hda–clamp–DNA complex interacts with ATP-DnaA molecules catalytically, simulating ATP hydrolysis to yield ADP-DnaA. This system is predominant in the inactivation of DnaA after replication initiation, and strongly represses over-initiation of replication. RIDA has been well documented elsewhere (Katayama et al., 2010; Skarstad and Katayama, 2013; Riber et al., 2016), and this review will focus on the two other DnaA regulatory systems.

DDAH (*datA*-dependent DnaA-ATP hydrolysis) regulates the inactivation of DnaA (Kasho and Katayama, 2013; Riber et al., 2016) and requires the non-coding, chromosomal DNA element *datA* (Kitagawa et al., 1996, 1998; **Figure 1**). The 262 bp minimal *datA* region has a specific DnaA-box cluster and a single IHF-binding site (IBS) (Kasho and Katayama, 2013; Kasho et al., 2017; **Figure 1B**). A *datA*–IHF complex forms after replication initiation, and stimulates formation of specific ATP-DnaA oligomers, and hydrolysis of DnaA-bound ATP, independently of RIDA.

DnaA-reactivating sequence (DARS) sites promote reactivation of DnaA (Fujimitsu et al., 2009). The *E. coli* genome has at least two DARS sites (*DARS1* and *DARS2*) (**Figure 1**), which contain specific DnaA-box clusters (**Figure 1B**), and promote oligomerization of ADP-DnaA, leading to the release of ADP (Fujimitsu et al., 2009; Kasho et al., 2014). The resultant nucleotide-free apo-DnaA preferentially dissociates from the complex, and binds ATP. Both *DARS1* and *DARS2* are required to sustain timely initiation *in vivo*. In particular, *DARS2* is activated by binding of IHF and Fis (a nucleoid-associated factor); IHF binding occurs temporarily before replication initiation (Kasho et al., 2014). The RIDA, DDAH, and DARS DnaA-regulating systems are all required for timely initiation of chromosomal replication.

This review provides an up-to-date detailed synopsis of the various factors and mechanisms involved, particularly from the perspective of the mechanisms involved in the regulation of the timely activation and inactivation of the initiator protein DnaA. Sections “Basic Features of *oriC* and DnaA and DnaA Complex on *oriC*” describe mainly the principal features of *oriC*, DnaA and *oriC*-DnaA complexes, which are based on progress gained over the last 30 years. Sections “DDAH System and DARS System” mainly describe recent developments regarding DDAH and DARS systems and the salient features of DnaA complexes at *datA* and DARS sites. These latter sections provide information on the mechanisms of functionally different DnaA-DNA complexes. Readers who are interested only in DDAH and DARS systems might skip the Section “DnaA Complex on *oriC*,” while readers who are interested only in *oriC*-DnaA complexes might skip the Sections “DDAH System and DARS System.”

## Basic Features of *oriC* and DnaA

### oriC

The *E. coli oriC* is a 245 bp DNA element located at 84.6 min of the circular chromosome, and composed of two functionally distinct regions: the DUE and the DnaA-oligomerization region (DOR) (**Figures 1A, 2A**). The DUE has three AT-rich 13-mer sequences (L, M, and R) with the consensus 5′-GATCTnTTnTTTT-3′, where the Watson and the Crick strands are T-rich and A-rich, respectively (Bramhill and Kornberg, 1988; Messer, 2002). An additional AT-rich region flanking the L sequence assists in DUE unwinding (Messer, 2002). When the DUE is unwound, the single-stranded T-rich DUE strand binds to DnaA oligomers that are bound to the DOR (Ozaki et al., 2008) (also see below). Stable binding of the single-stranded DUE (ssDUE) requires the presence of a region spanning at least the M and R sequences; 5′-TTGT-3′ and 5′-TTATT-3′ sequences are specifically required for the binding (Ozaki et al., 2008; **Figure 2B**). In *Bacillus subtilis oriC*, a repeating trinucleotide motif (DnaA-trio) with the consensus 5′-TA(A/G)-3′ is present in the AT-rich DUE and has been proposed to sustain ssDNA binding by direct interaction with DnaA (**Figure 2**; Richardson et al., 2016). As requirements of specific sequences in DUE are different, *E. coli* and *B. subtilis* may differ in their mechanisms for recognition of ssDUE by DnaA (**Figure 2B**).

The DOR is directly connected to the right edge of the DUE, and contains 12 DnaA boxes, which are present in both orientations (**Figure 2A**). The DOR can be subdivided into three structurally distinct sub-regions: left-half, middle, and right-half (Leonard and Grimwade, 2015; Shimizu et al., 2016). Of these, the left-half sub-region contains six DnaA boxes (R1, τ1, R5M, τ2, I1, I2) in one orientation, and the right-half sub-region contains five boxes (C3, C2, I3, C1, R4) in the opposite orientation, whereas the middle region contains one box (R2) in the right-half orientation (McGarry et al., 2004; Rozgaja et al., 2011; Ozaki and Katayama, 2012; Ozaki et al., 2012a; Shimizu et al., 2016; **Figure 2A**). DnaA complexes assembled on these different sub-regions have specific roles in DUE unwinding and DnaB-helicase loading.

DnaA boxes R1 and R4 (at the left and right ends of the DOR, respectively) match the consensus 5′-TTATnCACA-3′ and have the highest affinity among DOR boxes for DnaA (in both its ATP and ADP bound forms) (Messer, 2002; McGarry et al., 2004; Kawakami et al., 2005; **Figure 2A**). DnaA box R2 has basically moderate affinity, and ATP-DnaA more efficiently binds to this box present in DOR than ADP-DnaA (Messer, 2002; McGarry et al., 2004; Kawakami et al., 2005; Keyamura et al., 2009). All the other DnaA boxes (τ1–I2 and R4–C1 in the left and right halves, respectively) are clustered, and each individual DnaA box only has a low affinity (McGarry et al., 2004; Kawakami et al., 2005; Rozgaja et al., 2011). Although these low-affinity boxes have only moderate similarities to the DnaA-box consensus, ATP-DnaA-specific cooperative binding does occur. This binding depends on DnaA Arg285 for recognition of DnaA-bound ATP (Kawakami et al., 2005; Keyamura et al., 2007, 2009; Kaur et al., 2014; Sakiyama et al., 2017) (for details, see below). ATP-DnaA binding to box τ1 occurs in the absence of IHF binding, but not in the presence of IHF binding, probably because of steric interference (Kawakami et al., 2005; Sakiyama et al., 2017) (see below).

The left-half DOR has a specific binding motif for the nucleoid-associated, DNA-bending protein IHF (**Figure 2A**). The consensus of the IBS is 5′-(A/T)ATCAAnnnnTT(A/G)-3′ (Swinger and Rice, 2004). *In vitro*, IHF binding stimulates DUE unwinding and replication from *oriC*; another nucleoid-associated protein (HU) can substitute for IHF in these reactions (Hwang and Kornberg, 1992). IHF stimulates DnaA binding to moderate-affinity (R2) and low-affinity (R5M and I1-3) sites (Grimwade et al., 2000; McGarry et al., 2004), and promotes binding to ssDUE by DnaA that is complexed with the DOR (Ozaki and Katayama, 2012). Double mutations resulting in deficiency of both IHF and HU severely inhibit cell growth, and cause synthetic lethality at high temperatures (Kano and Imamoto, 1990). Although a Fis binding consensus sequence is present between the R2 and C3 boxes, mutant cells bearing a sequence defective in Fis binding and the results of *in vitro* replication systems suggest that binding of Fis to *oriC* is not an important element in the regulation of initiation (Margulies and Kaguni, 1998; Weigel et al., 2001).

### DnaA

**FIGURE 3:**
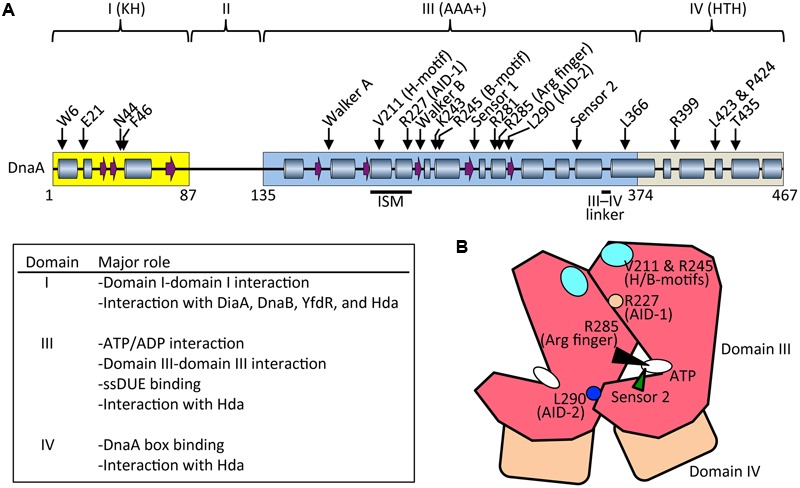
Basic features of DnaA. **(A)** Possible secondary structure of full length DnaA. Domains I, III, and IV are shown in yellow, blue, and gray boxes, respectively. α helices (cylinders) and β strands (arrows) were determined from the NMR structure of DnaA domain I–II (PDB: 2E0G; Abe et al., 2007) and the crystal structure of DnaA domain IV (PDB: 1J1V; Fujikawa et al., 2003), combined with a homology model of DnaA domain III generated using the crystal structure of *Thermotoga maritima* DnaA (PDB: 2Z4S; Ozaki et al., 2008) (SWISS-MODEL Repository: P03004). For clarity, extremely short helices in the homology model are omitted according to the crystal structure of *A. aeolicus* DnaA domain III (Erzberger et al., 2002). Representative motifs in the AAA+ fold and residues crucial for DnaA functions are indicated. Major roles of domains I, II, and IV are summarized. Arg finger, arginine finger (Arg285). **(B)** Schematic presentation of domain III (*red*) and IV (*brown*). Important motifs and residues for DnaA–DnaA interaction and ATP recognition/hydrolysis in DnaA complexes are indicated. ATP, white oval; arginine finger Arg285, black arrowhead; AID-1 Arg227, orange circle; AID-2 Leu270, blue circle; sensor 2, green arrowhead; and H/B-motifs Val211 and Arg245, cyan oval.

DnaA is a 52.5 kDa basic protein composed of 473 amino acids in four domains (Ozaki and Katayama, 2009; Kaguni, 2011; **Figure 3A**). The largest (domain III) has an AAA+ ATPase fold that contains Walker A and B ATP/ADP-binding/hydrolysis motifs, motifs for domain III–domain III head-to-tail interaction, and motifs for ssDUE binding. Domain III AAA+ sensor 1 motifs and N-linker motif contribute to an extraordinarily high affinity for ATP/ADP (*K*_d_ = 10–100 nM) (Kawakami et al., 2006; Ozaki et al., 2012b). ATP binding causes conformational changes in DnaA, and structural differences between ATP-DnaA and ADP-DnaA are thought to occur in domains II and III (Saxena et al., 2015). Lys178, an essential residue within the Walker A motif of DnaA, is acetylated specifically in stationary-phase cells (Zhang et al., 2016).

ATP-DnaA forms oligomers at *oriC* more efficiently than ADP-DnaA. The arginine finger motif (Arg285), ATP-DnaA-specific interactive locus for DUE unwinding (AID) motifs (Arg227, Leu290), box VII motif (Arg281), and Lys243 are required for functional domain III–domain III interactions (Felczak and Kaguni, 2004; Kawakami et al., 2005; Ozaki et al., 2008, 2012a; **Figure 3**) (see section Structure of the DnaA–IHF–*oriC* Complex for DUE Unwinding and DnaB Loading). In particular, Arg285 Arg finger is important for the ATP-dependent activation of DnaA complexes on *oriC* (Kawakami et al., 2005) and is exposed on the side opposite to that of the ATP/ADP-binding site of domain III (**Figure 3B**). Whereas DNA-free DnaA is monomeric in solution, domain III enables DnaA to self-oligomerize in an ATP-dependent, head-to-tail manner via interactions between bound ATP and Arg285 of adjacent protomers, resulting in the binding of multiple DnaA protomers to *oriC* (Kawakami et al., 2005; Ozaki et al., 2008).

During unwinding of DUE by DnaA, hydrophobic (H) and basic (B) motifs (Val211 and Arg245, respectively), which are highly conserved in DnaA orthologs, have a crucial role in binding to the T-rich ssDUE strand (Ozaki et al., 2008; Ozaki and Katayama, 2012; Sakiyama et al., 2017; **Figure 3**) (see Section Structure of the DnaA–IHF–*oriC* Complex for DUE Unwinding and DnaB Loading). The H and B motifs are located in helices between the Walker A and B motifs and the Walker B and sensor 1 motifs, respectively (Ozaki and Katayama, 2009; **Figure 3A**). Whereas typical AAA+ proteins have only a single α helix between Walker A and B motifs, DnaA-related proteins (collectively termed the replication initiator clade) have two α helices in tandem, and the H motif is located on one of the two, giving a structure called an initiator/loader-specific motif (Iyer et al., 2004; Dueber et al., 2007; Mott et al., 2008; Ozaki et al., 2008; Ozaki and Katayama, 2009).

Certain residues in DnaA have distinct roles in its ATPase activity. Sensor 2 Arg334 is important for the intrinsic ATPase activity of DnaA, RIDA, and DDAH, probably via an interaction between the γ phosphate of ATP and Arg334. However, this residue is dispensable for the formation of functional initiation complexes on *oriC* (Sekimizu et al., 1987; Nishida et al., 2002; Kasho and Katayama, 2013; **Figure 3**). Arginine finger Arg285 is essential for intrinsic ATPase activity and for regulation by DDAH, but not by RIDA (Kawakami et al., 2005; Kasho and Katayama, 2013). Box VII Arg281 represses intrinsic ATPase activity and is dispensable for RIDA, but is required for DDAH (Kawakami et al., 2005; Kasho and Katayama, 2013) (see below).

The DnaA C-terminal domain IV has a typical helix-turn-helix (HTH) motif and binds with sequence specificity to DnaA boxes (Erzberger et al., 2002; Obita et al., 2002; Fujikawa et al., 2003; Yoshida et al., 2003; **Figure 3A**). This binding causes DNA bending by about 20°. An α helix consisting of amino-acid residues 434–451 is part of the HTH motif that is inserted in the major groove of the 9 bp DnaA box, recognizing the 3′ portion (5′-TnCACA-3′) of the consensus sequence (Fujikawa et al., 2003). Arg399, which is located in the turn (or loop) region of the HTH motif, is inserted in the minor groove of the DnaA box, recognizing the 5′ portion (5′-TTA-3′) of the consensus sequence (Fujikawa et al., 2003). In consistent, DnaA T435M and DnaA R399A are defective in DnaA-box binding (Sutton and Kaguni, 1997; Blaesing et al., 2000). The short linker connecting domains III and IV is a structurally flexible loop that enables swiveling of domain IV (Erzberger et al., 2002), and the results of molecular dynamics simulation indicate that it is important in the assembly of DnaA oligomers on *oriC* (Shimizu et al., 2016; **Figure 3A**). Leu366 located in an α helix downstream of the short linker is required for a conformational change in DnaA complexes constructed on *oriC* (Garner and Crooke, 1996; Saxena et al., 2011; **Figure 3A**). In addition, Leu423 and Pro424 contribute to the Hda binding that is crucial for RIDA (Keyamura and Katayama, 2011).

The N-terminal domain I of DnaA has important roles in protein–protein interaction. Weak domain I–domain I binding depends on Trp6, which contributes to DnaA self-oligomerization (Sutton et al., 1998; Simmons et al., 2003; Felczak et al., 2005; **Figure 3A**). Glu21 and Phe46 form a binding site for both DnaB helicase and DiaA protein (Abe et al., 2007; Keyamura et al., 2009). Phe46 contributes more to DiaA binding than Glu21, and is also required for binding to YfdR protein, a potential inhibitor of DnaA–DiaA or DnaA–DnaB interactions that is encoded by a cryptic prophage (Noguchi and Katayama, 2016). Asn44 is thought to interact with Hda to facilitate functional interaction between DnaA domain III and Hda during RIDA (Su’etsugu et al., 2013). Domain I also binds to HU, Dps (DNA protection during starvation protein), and ribosomal L2 protein, but the residues involved in these interactions have not yet been identified (Chodavarapu et al., 2008a,b, 2011). In addition, DnaA domain I contains a K-homology domain, which enables binding to ssDNA and RNA (Abe et al., 2007). However, domain I has only slight activity in ssDUE binding *in vitro* (Abe et al., 2007), and notably, DnaA with deletion of domains I and II retains full DUE-unwinding activity *in vitro* (Sutton et al., 1998), which indicates that domain III plays a predominant role in ssDUE binding sustaining DUE unwinding (Ozaki et al., 2008; Ozaki and Katayama, 2012; Sakiyama et al., 2017). Domain II is an unstructured linker and the least-conserved domain among DnaA orthologs in eubacterial species (Abe et al., 2007; Nozaki and Ogawa, 2008; **Figure 3A**).

## DnaA Complex on *oriC*

### The Role of DiaA

The 196 amino acid DiaA protein forms a homotetramer in which each protomer has a DnaA-binding site (Ishida et al., 2004; Keyamura et al., 2007, 2009). DiaA stimulates ATP-DnaA assembly on *oriC* and the unwinding of DUE. A single DiaA tetramer can bind multiple DnaA molecules (theoretically up to four; experimentally at least three), potentially leading to the linkage effect for stimulating cooperative binding. With the linkage effect, it has been demonstrated that, if two proteins each with a weak affinity for DNA are stably connected by a linker molecule, the affinity of the linked molecule for DNA is increases drastically (theoretically by up to 10^3^- to 10^5^-fold) because it permits binding between the protein and multiple points on the DNA (Zhou, 2001; Stauffer and Chazin, 2004). A mutant that encodes a variant of DiaA with constitutive binding of just two DnaA protomers is defective in stimulation of DnaA assembly on *oriC* and DUE unwinding (Keyamura et al., 2007). The sequence of the *diaA* gene is widely conserved in the genomes of eubacterial species (Keyamura et al., 2007).

The DiaA-binding site is located in domain I of DnaA (and includes residues Glu21 and Phe46), and this site also binds DnaB helicase, as described in Section DnaA (Abe et al., 2007; Keyamura et al., 2009; **Figure 3A**). Both overproduction of DiaA and deletion of the *diaA* gene moderately inhibit initiation *in vivo* (Ishida et al., 2004; Flåtten et al., 2015). DiaA therefore has a positive effect on initiation through stimulation of DnaA assembly, and a negative effect probably via its ability to inhibit DnaA–DnaB binding. DiaA that is bound to the DnaA assembly on *oriC* is thought to dissociate (enabling DnaB binding) at (or after) the time of DUE unwinding, by an unknown mechanism (Keyamura et al., 2009). Regulation of the inhibitory activity of DiaA has not yet been determined.

### Structure of the DnaA–IHF–*oriC* Complex for DUE Unwinding and DnaB Loading

**FIGURE 4:**
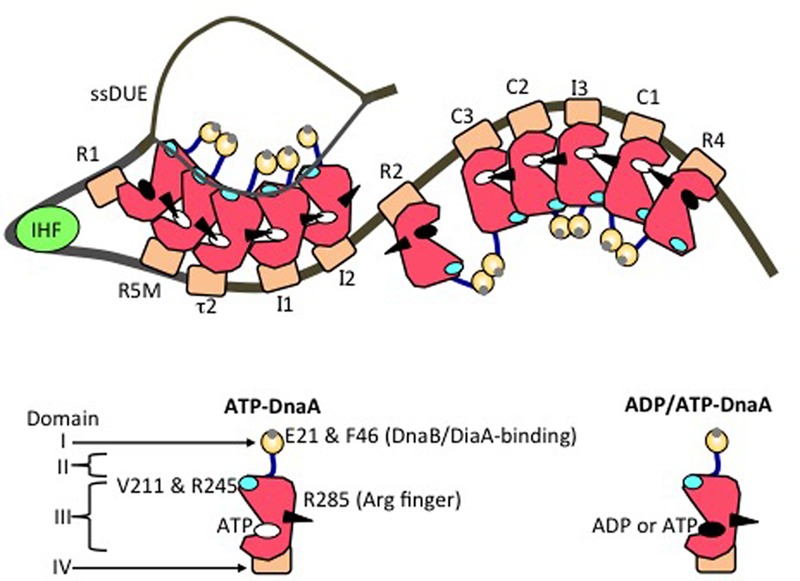
Model of an unwound state of the initiation complex. DnaA domains I (*orange*), II (*blue*), III (*red*), and IV (*brown*) are schematically presented. A small gray circle on domain I shows a patch including Glu21 and Phe46 (for binding of DiaA and DnaB helicase). A black arrowhead, cyan oval, white oval, and black oval on domain III represent the arginine finger Arg285, H/B motifs, ATP, and either ADP or ATP, respectively. IHF is shown as a light green circle. DnaA-box positions are labeled. On the left-half and right-half DnaA-oligomerization regions, head-to-tail DnaA-oligomer sub-complexes assemble, with interactions between ATP and Domain III Arg285. The T-rich ssDUE strand interacts with the DnaA H/B motifs in the left-half sub-complex.

The question of the regularity and directionality of DnaA boxes in *oriC* has been resolved by the identification of low-affinity DnaA boxes, and by the correction of previously obtained information. The left-half DOR is now known to contain only DnaA boxes with a leftward directionality (R1, τ1, R5M, τ2, I1, I2), whereas the middle region and right-half DOR contain only DnaA boxes with a rightward directionality (R2, C3, C2, I3, C1, R4) (**Figure 2A**). As explained above, head-to-tail binding of domain III leads to ATP-DnaA-dependent assembly of sub-complexes with opposite orientations in the left-half and right-half DORs (Kawakami et al., 2005; Noguchi et al., 2015; Shimizu et al., 2016; Sakiyama et al., 2017; **Figures 3, 4**).

In DnaA sub-complexes, nucleotides that are associated with R1 box- and R4 box-bound DnaA protomers are located at the outer edges of the DOR, so that those cannot interact with DnaA protomers bound to other DOR sites (**Figures 2A, 4**). Thus, even when ADP-DnaA is bound to R1, DUE-unwinding activity is fully sustained (Noguchi et al., 2015). ATP-DnaA assembly at the left-half DOR fundamentally depends on ATP–DnaA binding to the low-affinity R5M box (but not to the R1 box) and is stimulated by R1-bound DnaA (Sakiyama et al., 2017). Cooperative binding of ATP-DnaA molecules would operate effectively in the region from the R5M box to the I2 box (Sakiyama et al., 2017). Also, when ADP-DnaA is bound to the R4 box, assembly of the DnaA sub-complex on the right-half DOR is fully sustained with ATP-DnaA (Noguchi et al., 2015). ATP-DnaA assembly to the low-affinity sites in the right-half DOR depends on DnaA binding to the R4 box (Noguchi et al., 2015).

Evidence suggests that domain III of R2-bound DnaA does not interact with DnaA protomers bound to I2 or C3 (Rozgaja et al., 2011; Shimizu et al., 2016), as expected by the long spacing between R2 and I2 (9 bp) and between R2 and C3 (20 bp) (**Figure 2A**). Each box in the τ1-R5M-τ2-I1-I2 region and the R4-C1-I3-C2-C3 region is separated by 2–4 bp. However, domain I of R2-bound DnaA might interact with that of I2-bound or C3-bound DnaA, simulating cooperative DnaA binding (Miller et al., 2009; Ozaki and Katayama, 2012; Shimizu et al., 2016; **Figure 4**).

The existence of nucleotide-independent activities of DnaA bound to R1, R4, and R2 is consistent with data from *in vivo* dimethyl-sulfate footprint analysis, which suggest that DnaA binding to these boxes is stable throughout the cell cycle (Samitt et al., 1989; McGarry et al., 2004; Kawakami et al., 2005). By contrast, in *in vivo* experiments with DnaA fused to enhanced yellow fluorescent protein, results from photobleaching and high-resolution microscopy suggest that, overall, DnaA complexes bound to *oriC* are rapidly turned over with a time scale of a few seconds (Schenk et al., 2017). These results are consistent with the unstable binding of DnaA to low-affinity sites. Such dynamic features might be important for timely assembly of ATP-DnaA on *oriC* after the DARS system has increased in total level of ATP-DnaA.

The left-half ATP-DnaA sub-complex possesses full activity for DUE unwinding and ssDUE binding (Ozaki and Katayama, 2012; **Figure 4**). In addition to the Arg finger Arg285, AID1 (Arg227) and AID2 (Leu290) motifs, which are highly conserved in DnaA orthologs, are thought to be located at the domain III–domain III interface and to contribute to ATP–DnaA-specific interactions and the establishment of DnaA oligomers within the left-half DOR that are competent for DUE unwinding (Ozaki et al., 2012a; **Figure 3B**). Lys243 also contributes to oligomerization of ATP-DnaA on *oriC* and DUE unwinding (Ozaki et al., 2008). Also, Lys243 can be acetylated *in vivo*. Although the significance of this is unknown, it moderately affects DnaA binding to *oriC in vitro* (Li et al., 2017). Molecular dynamics simulation shows that sharp DNA bending (>120°) caused by IHF binding enables domain III–domain III interaction between DnaA protomers bound to R1 and R5M in the absence of DnaA–τ1 binding (Shimizu et al., 2016; **Figure 4**). Consistently, *in vitro* experiments indicate that DnaA–τ1 interaction is inhibited by IHF binding (Sakiyama et al., 2017).

DUE unwinding depends on temperature and superhelicity of *oriC* DNA, which contribute to destabilization of the duplex state of the DUE (Baker and Kornberg, 1988; Sekimizu et al., 1988). In addition, the unstable ssDUE binds to, and is stabilized by, DnaA domain III, enabling loading of DnaB helicase to the unwound region (Ozaki et al., 2008). In *E. coli* and *B. subtilis*, only a specific strand (the T-rich strand in *E. coli*) binds to DnaA (Ozaki et al., 2008; Richardson et al., 2016). Also, in *E. coli*, the H/B-motif residues of domain III are required for ssDUE binding (Ozaki et al., 2008; Sakiyama et al., 2017). This binding mode is consistent with that seen in the co-crystal structure of the poly-A ssDNA-bound DnaA domain III ortholog from the hyperthermophilic bacterium *Aquifex aeolicus* (Duderstadt et al., 2011).

Notably, when multiple DnaA molecules bind to the DOR, ssDUE binding by DnaA increases dramatically and is much higher than that of DOR-unbound DnaA molecules (Ozaki et al., 2008; Ozaki and Katayama, 2012; Sakiyama et al., 2017). ssDUE directly binds to domain III of DnaA protomers that have associations through domain IV with the left-half DOR (Ozaki et al., 2008; Ozaki and Katayama, 2012; Sakiyama et al., 2017; **Figure 4**). In particular, domain III of the R1- and R5M-bound DnaA protomers play predominant roles in ssDUE binding for replication initiation (Sakiyama et al., 2017). These and the other observations mentioned above support the idea that ssDUE directly binds to R1- and R5M-bound DnaA protomers via sharp DNA bending by IHF, which stimulates ssDUE recruitment to those DnaA molecules. This is referred to as the ssDUE-recruitment mechanism (**Figure 4**; Ozaki et al., 2008; Ozaki and Katayama, 2012; Noguchi et al., 2015; Shimizu et al., 2016; Sakiyama et al., 2017). This reasonably explains the role of IHF and dispensability of ATP of R1-bound DnaA in DUE unwinding, and the striking stimulation of ssDUE binding by DnaA-oligomer assembly on the DOR, as well as the strict requirements in DUE unwinding for the spacing between the DUE and the DnaA R1 box and between the DnaA R1 box and the IBS, and the requirement for H/B motifs of the DOR-bound DnaA for ssDUE binding (**Figure 4**) (for details, see Sakiyama et al., 2017).

Multiple DnaA molecules bound to the DOR are required for binding of DnaB helicase which is a homohexamer (Abe et al., 2007; Keyamura et al., 2009; Ozaki and Katayama, 2009). Multiple DnaA domain I molecules of the DOR-bound DnaA complexes would be allied so as to stably bind DnaB helicases by using multiple binding points (**Figure 4**). In consistent, Box VII Arg281 stabilizes DnaA oligomers assembled on the DOR, and is required for DnaB-helicase loading, but not for DUE unwinding (Felczak and Kaguni, 2004). This suggests that stability of DnaA complexes is more important for DnaB-helicase loading than for DUE unwinding.

In addition to the basic DnaB-binding/loading activity of the left-half-DOR DnaA sub-complex, the right-half-DOR DnaA sub-complex stimulates DnaB binding/loading (Ozaki and Katayama, 2012; **Figures 2A, 4**). This activity might result in binding of two DnaB helicases, one to each of the DnaA sub-complexes, enabling bi-directional replication (Ozaki and Katayama, 2012; Noguchi et al., 2015; **Figure 4**). The spacing between the R2 and C3 boxes is important for efficient DnaB loading, suggesting coordination of the left and right DnaA subcomplexes in a DnaB loading process (Shimizu et al., 2016; **Figure 4**).

## DDAH System

### Function

**FIGURE 5:**
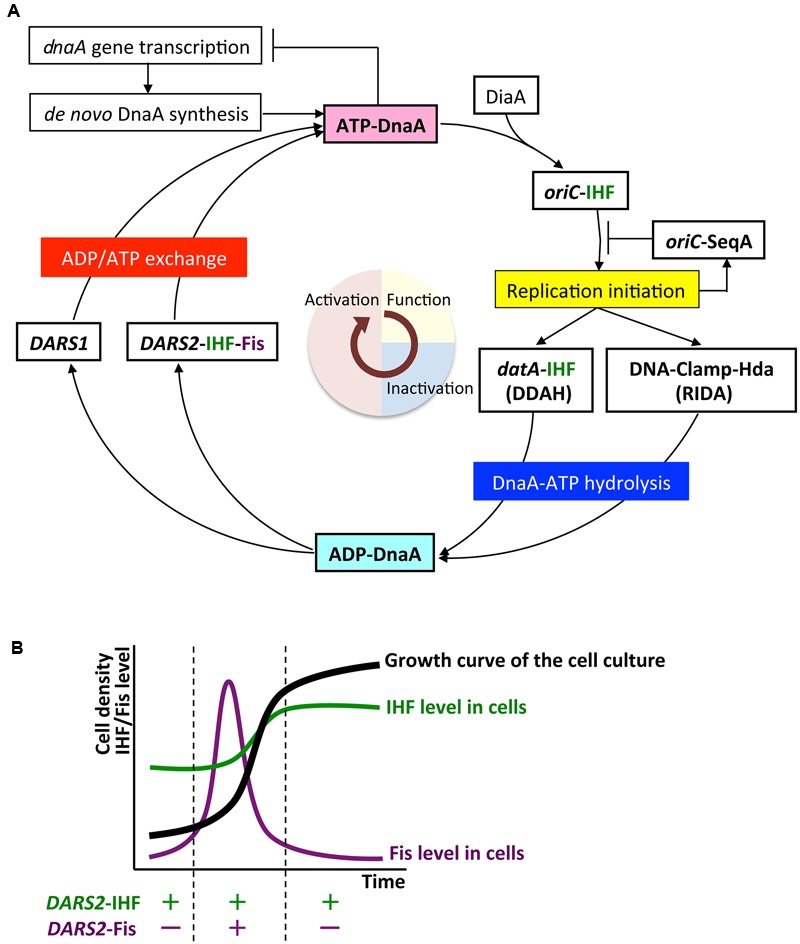
The regulatory cycle of DnaA. **(A)** Schematic presentation of the regulatory cycle of DnaA. ATP-DnaA forms oligomers on *oriC* with the aid of integration host factor (IHF) and DnaA interacting protein DiaA, and initiates replication (period in light yellow). After initiation, ATP-DnaA is converted to replication-inactive ADP-DnaA (RIDA) and *datA*-dependent DnaA-ATP hydrolysis (DDAH) systems (period in light blue). RIDA involves Hda protein and the clamp subunit of DNA polymerase III holoenzyme, and DDAH involves the *datA* locus and IHF. DnaA-reactivating sequence (*DARS1* and *DARS2*) loci stimulate nucleotide exchange of ADP-DnaA and regenerate ATP-DnaA (period in light red). IHF binds to specific sites in *oriC, datA*, and *DARS2* in a cell-cycle-coordinated manner. Fis binds to *DARS2* in log-phase cells. **(B)** Growth-phase coordination of regulation of Fis expression. The cellular level of Fis varies through the growth phases, and is much higher in exponential-phase cells (enabling Fis binding to *DARS2*) than in stationary-phase cells. By contrast, the cellular level of IHF is highest in stationary-phase cells.

*datA*-dependent DnaA inactivation has been termed DDAH. Three hundred 9-mer DnaA box consensus sequences are widely distributed throughout the *E. coli* chromosome (Tesfa-Selase and Drabble, 1992; Roth and Messer, 1998; Hansen et al., 2007). Some of these sites regulate the activity of DnaA for replication initiation, whereas some of others function for transcriptional regulation by DnaA (Kitagawa et al., 1996; Messer and Weigel, 1997; Speck et al., 1999; Olliver et al., 2010). The *datA* locus includes a DnaA-box cluster and was originally identified as a potent DnaA-binding locus that can repress untimely replication initiation (Kitagawa et al., 1996; Ogawa et al., 2002; Nozaki et al., 2009; **Figure 1A**). DnaA binding at *datA* is stimulated by IHF (Nozaki et al., 2009); the *datA*–IHF complex stimulates hydrolysis of DnaA-bound ATP independently of RIDA to produce ADP-DnaA (Kasho and Katayama, 2013; Kasho et al., 2017; **Figure 5A**).

Several lines of *in vivo* evidence point to the important role of the *datA*–IHF complex in the repression of untimely replication initiation. Deletion of *datA* or IHF genes (*ihfA* or *ihfB*) increases cellular levels of ATP-DnaA, resulting in higher levels of ATP-DnaA than in wild-type cells and untimely replication initiation without cell growth inhibition (Kitagawa et al., 1996; Nozaki et al., 2009; Kasho and Katayama, 2013). As inhibition of RIDA causes severe over-initiation of replication and arrest of cell growth (Kato and Katayama, 2001; Nishida et al., 2002; Fujimitsu et al., 2008), DDAH is thought to play a supportive role in RIDA. Also, in DDAH-deficient mutants, replication initiation is not completely repressed in the presence of rifampicin, an inhibitor of transcription and replication initiation (von Freiesleben et al., 2000; Morigen, Skarstad and Molina, 2005), probably due to elevated levels of ATP-DnaA.

### Structure of *datA*

The minimal *datA* for DDAH function is 183 bp containing high-affinity DnaA boxes 2 and 3, low-affinity DnaA box 7 and a single IBS, which are all essential for efficient ATP–DnaA binding and DDAH activity (Kitagawa et al., 1998; Ogawa et al., 2002; Nozaki et al., 2009; Kasho and Katayama, 2013; Kasho et al., 2017; **Figure 1B**). All the essential DnaA boxes have the same directionality, suggesting the importance of head-to-tail ATP–DnaA interactions. The results of mutation analyses indicate that, as in the *oriC* DORs, the orientations and interval lengths of the *datA* DnaA boxes 2 and 3 and the IBS are optimized for repression of untimely initiations through DDAH (Nozaki et al., 2009; Kasho and Katayama, 2013). The *datA* DnaA box 4 stimulates DnaA assembly and DnaA-ATP hydrolysis *in vitro*, but is not essential for initiation regulation *in vivo* (Ogawa et al., 2002; Kasho and Katayama, 2013; Kasho et al., 2017; **Figure 1B**). Unidentified factor(s) might stimulate DnaA assembly *in vivo* even in the absence of DnaA box 4 (see below).

### Mechanism

**FIGURE 6:**
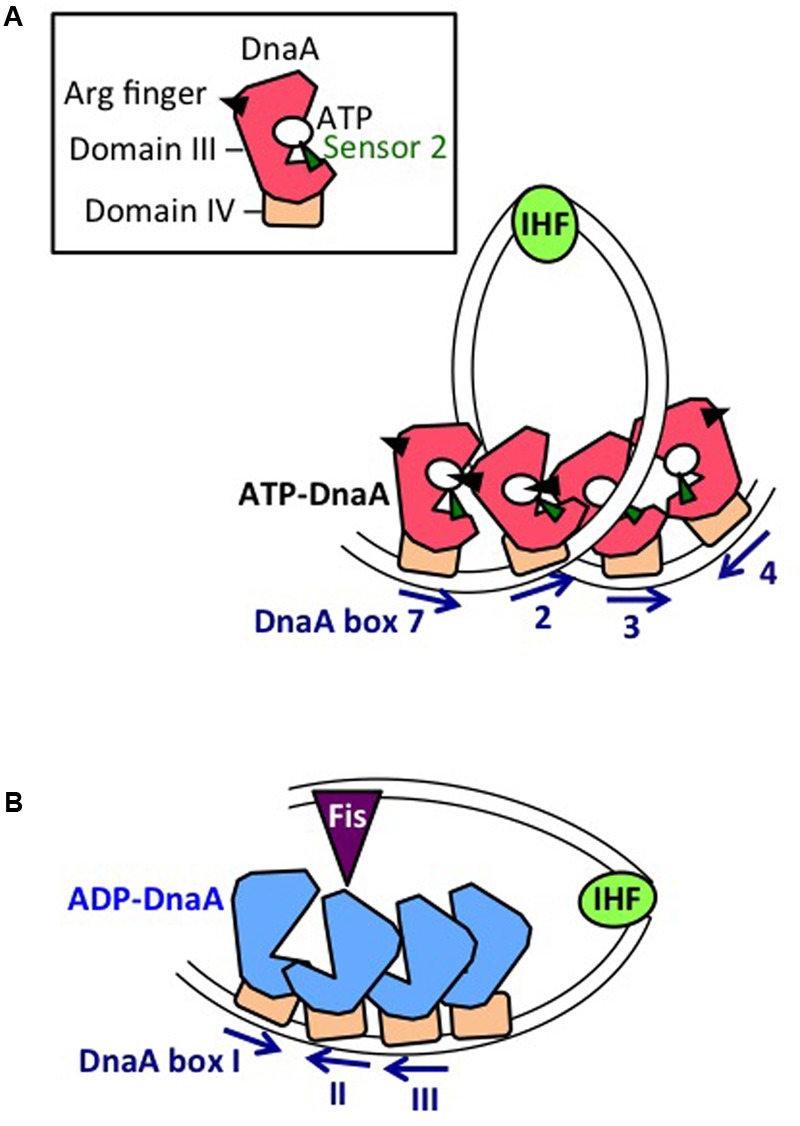
Mechanistic view of DnaA regulation by *datA* and *DARS2*. **(A)** Mechanistic model of *datA*-dependent DnaA-ATP hydrolysis (DDAH). Oligomers of ATP-DnaA (with domain III shown as a red polygon, and domain IV as an brown square) assemble on the *datA* minimal region containing DnaA boxes 7, 2, and 3. Sharp DNA bending by IHF could stimulate contact between DnaA bound at boxes 2 and 3. Cooperative ATP-DnaA binding on *datA* DnaA box 7 might induce conformational change of the DnaA to activate DDAH. **(B)** Mechanistic model of *DARS2*-mediated DnaA activation. Oligomers of ADP-DnaA (with domain III shown as a blue polygon, and domain IV as an brown square) assemble on the *DARS2* core region. IHF and Fis bind to specific sites in the *DARS2* regulatory region, and the resultant complex stimulates conformational changes in the core-bound DnaA oligomer, to induce structural changes in DnaA and ADP dissociation. Nucleotide-free apo-DnaA dissociates from the core region and binds ATP (which is abundant in the cytosol), yielding ATP-DnaA.

In the *datA* region, oligomers containing three ATP-DnaA molecules form on and around the core DnaA boxes and higher oligomers form depending on IHF (Kasho and Katayama, 2013; Kasho et al., 2017; **Figure 6A**). IHF binding causes DNA looping, which would promote interaction between the box 2-bound DnaA and the box 3-bound DnaA, thereby stabilizing DnaA complexes in a cooperative manner for the activation of DnaA-ATP hydrolysis. The results of biochemical analyses using mutant DnaA proteins provide evidence that, in *datA*–DnaA complexes, DnaA–DnaA interactions depend on AAA+ domain III arginine finger Arg285, box VII Arg281, and AID2 Leu290, and stimulation of ATP hydrolysis depends on AAA+ sensor 2 motif Arg334 (Kasho and Katayama, 2013; Kasho et al., 2017). DnaA AID-2 Leu290 might facilitate dissociation of the resultant ADP-DnaA, which is unstable in *datA*, thereby enabling catalytic reaction of DDAH (Kasho et al., 2017). Overall, these requirements for specific residues are similar to those of the initiation complex, except for the crucial role of sensor 2 Arg334 in ATP hydrolysis. This seems reasonable because both DDAH complexes and the initiation complex are constructed mainly from head-to-tail DnaA–DnaA interactions (**Figures 3B, 6A**).

The stimulatory role of IHF in DDAH cannot be replaced by HU, which is a structural homolog of IHF and a widely conserved nucleoid-associated protein in most bacteria, although IHF can be functionally replaced by HU in an *in vitro* reconstituted system of replication initiation from *oriC* (Hwang and Kornberg, 1992; Dillon and Dorman, 2010; Kasho and Katayama, 2013). Like IHF, HU induces sharp DNA bending, but unlike IHF, HU binds to DNA without sequence specificity. At *datA* and *oriC*, the IBS is located between DnaA boxes, but the inter-DnaA box region in *datA* (i.e., between box 2 and box 3) is longer than that (i.e., between R1 box and R5M box) at *oriC* (**Figures 1B, 2A**), suggesting that a functional conformational change in *datA* for activating DDAH is strongly dependent on specific DNA bending at the *datA* IBS induced by IHF binding.

### Regulation

#### Coupling of *datA* Dosage (Copy Number) with Replication

*datA* is located at 94.7 min (4.39 Mb position) in the *E. coli* genome, near to *oriC* (84.6 min, 3.92 Mb position), which means that *datA* is duplicated soon after replication initiation, resulting in a temporary higher dosage (copy number) of *datA* per cell. This is thought to be important for the repression of untimely initiations under certain growth conditions (Kitagawa et al., 1996; **Figure 1A**). In agreement with these observations, increasing the *datA* copy number via a multi-copy plasmid negatively impacts replication initiation and cell growth (Morigen et al., 2003; Kasho et al., 2017). Conversely, translocation of *datA* to the replication-termination locus *terC* at 34.6 min, which is >2 Mb apart from *oriC* (**Figure 1A**), does not result in a temporary increase in its copy number until replication is nearly completed, allowing untimely initiations in nutrition-rich medium, similar to those observed in *datA*-deleted cells (Kitagawa et al., 1998; Frimodt-Møller et al., 2015). The *datA* copy number may also be important for the regulation of cell division (Morigen et al., 2014). Notably, in *B. subtilis* and *Streptomyces coelicolor*, chromosomal DnaA-box clusters analogous to *datA* repress untimely replication initiation (Smulczyk-Krawczyszyn et al., 2006; Okumura et al., 2012), suggesting that the DnaA regulation by *datA* is a conserved feature of most eubacterial species.

#### Cell-Cycle-Dependent Binding of IHF

The activation of DDAH and RIDA reduces the ATP-DnaA level and represses untimely initiations (Kasho and Katayama, 2013). Regulation of IHF binding to *datA* is essential for the timely activation of DDAH. Cell-cycle analysis of *datA*–IHF binding indicates that IHF dissociates from *datA* before replication initiation and temporarily binds to *datA* after initiation (Kasho and Katayama, 2013). As both IHF binding to and dissociation from *datA* occur during on-going chromosome replication, DDAH is suggested to be regulated by a mechanism that couples certain cell cycle events to *datA*–IHF binding. Conversely, RIDA is activated by DNA replication-coupled feedback (Katayama et al., 2010; Kasho and Katayama, 2013)

The transcription inhibitor rifampicin might inhibit timely dissociation of *datA*–IHF complexes (Kasho and Katayama, 2013). As IHF is abundant in exponentially growing cells (Ali Azam et al., 1999), a specific factor inhibiting *datA*–IHF binding might be required to be transcribed at a particular moment in the cell cycle. DDAH activity is inhibited by translocation of the *datA* sequence to a highly transcribed region (Frimodt-Møller et al., 2016), suggesting that transcription through *datA* might inhibit *datA*–IHF binding. Consistently, the essential core region of *datA* (i.e., box 7-box 2-IBS-box 3) is located at an intergenic region downstream of the *glyVXY* operon and *queG* gene (Kitagawa et al., 1998; Nozaki et al., 2009; Kasho et al., 2017; **Figure 1B**). It remains to be determined whether transcription of these genes oscillates in a coordinated manner during the cell cycle.

#### Effects of DNA Superhelicity and Nutrition

DDAH is suggested to regulated also by DNA supercoiling. *In vitro* experiments indicate that negative DNA supercoiling, which is modulated by DNA topoisomerases such as DNA gyrase and by nucleoid protein binding (Travers and Muskhelishvili, 2005), stabilizes ATP-DnaA oligomerization and IHF binding on *datA* and increases DDAH activity twofold compared with *datA* activity on relaxed DNA (Kasho et al., 2017). Modulation of negative supercoiling by novobiocin, a DNA-gyrase inhibitor, decreases the function of *datA* on plasmid DNA for repressing replication initiation (Kasho et al., 2017), suggesting supercoiling-dependent regulation of DDAH *in vivo*. Negative supercoiling is predicted to increase under osmotic-stress conditions (Higgins et al., 1988), which might affect *datA* activity for repressing initiation.

Nutrition-dependent regulation of DDAH has been suggested. Untimely initiations in *datA*-null cells are more severely exhibited in nutrient-poor medium containing acetate or glucose than in rich medium containing glucose-casamino acid or LB (Kitagawa et al., 1998; Flåtten et al., 2015). Chromosome-conformation-capture analysis has revealed that *oriC* can physically interact with *datA* under conditions of replication stress induced by serine hydroxamate, which causes amino-acid starvation and inhibits replication initiation (Cagliero et al., 2013). Free DnaA molecules present around *oriC* could efficiently interact with *datA* under poor nutrition conditions. Conversely, in rich medium, the *datA* dosage could be important for the regulation of DDAH. As described above, *datA* translocation to *terC* causes excess initiations in rich medium, but not in poor medium (Kitagawa et al., 1998).

## DARS System

### Function

In *E. coli*, ATP-DnaA is produced by binding of apo-DnaA to ATP, which is abundant in the cytosol (Bochner and Ames, 1982). Replication initiation occurs when the cellular level of ATP-DnaA reaches a peak that is high enough to enable assembly on *oriC* (Kurokawa et al., 1999). At least three mechanisms for production of apo-DnaA have been characterized: *de novo* DnaA synthesis; nucleotide dissociation from ADP-DnaA by acidic phospholipids in the cell membrane; and a mechanism involving specific chromosomal DNA elements termed DARS sites (**Figure 1**).

In *de novo* synthesis, newly translated apo-DnaA binds to ATP in the cytosol (**Figure 5A**). In addition to autoregulation by DnaA (Speck et al., 1999), transcription of *dnaA* is down-regulated by the SeqA-Dam system immediately after initiation (Campbell and Kleckner, 1990; Bogan and Helmstetter, 1997). This would contribute to the reduction in *de novo* DnaA synthesis (i.e., production of ATP-DnaA) immediately after initiation and maintain coordination between replication initiation and the cell cycle (Riber and Løbner-Olesen, 2005).

The *E. coli* cell membrane contains acidic phospholipids such as cardiolipin and phosphatidylglycerol that can promote dissociation of ADP or ATP from DnaA by interaction with DnaA domain III. These phospholipids can convert ADP-DnaA to ATP-DnaA *in vitro* (Saxena et al., 2013). In mutant cells with reduction of levels of acidic phospholipids, initiation from *oriC* is repressed (Fingland et al., 2012). The possibility that acidic phospholipids regulate DnaA activity during the cell cycle deserves to be further investigated.

The *DARS1* and *DARS2* chromosomal DNA sequences promote dissociation of ADP from ADP-DnaA, stimulating nucleotide exchange and increasing levels of ATP-DnaA, thereby stimulating replication initiation (Fujimitsu et al., 2009; Kasho et al., 2014; **Figures 1, 5A**). *In vitro, DARS1* can promote ADP dissociation of ADP-DnaA without additional factors, whereas *DARS2* has little activity by itself, but is substantially activated by IHF and Fis, which binds site-specifically and bends DNA by <60° (Fujimitsu et al., 2009; Kasho et al., 2014; **Figure 1B**). In cells, *DARS1* and *DARS2* both have stimulatory roles in ATP-DnaA production and replication initiation, and the deletion of either or both delays initiation (Fujimitsu et al., 2009; Kasho et al., 2014). Deletion of *DARS2* inhibits timely initiation more severely than deletion of *DARS1*, causing asynchronous initiations, and consistently, oversupply of *DARS2* has a greater effect than oversupply of *DARS1* on stimulation of extra initiations, which can cause inhibition of cell growth (Fujimitsu et al., 2009; Kasho et al., 2014). Deletion of *DARS2*, substitution of the IBS or Fis-binding site (FBS), or deletion of Fis gene decreases the cellular ATP-DnaA level more than deletion of *DARS1* under RIDA-deficient conditions (Fujimitsu et al., 2009; Kasho et al., 2014; **Figure 1B**). These observations suggest that the complex formed by *DARS2*, IHF, and Fis is important for increasing the level of ATP-DnaA and determining initiation timing, and that *DARS1* might maintain basal levels of ATP-DnaA.

### Structure of *DARS1* and *DARS2*

A common structural feature of *DARS1* (101 bp) and *DARS2* (455 bp) is the presence of a core region containing three DnaA boxes (DnaA boxes I, II, and III): the spacing between these boxes is the same at both *DARS1* and *DARS2*, and box I has the opposite orientation to boxes II and III (**Figure 1B**). In addition to the core regions, *DARS1* and *DARS2* have regulatory regions of different lengths. The ∼50 bp regulatory region of *DARS1* enhances DnaA-ADP-dissociation activity *in vitro*, but its role *in vivo* has not been determined (Fujimitsu et al., 2009). The ∼400 bp *DARS2* regulatory region contains the IBS and FBS, which stimulate ADP-DnaA assembly on *DARS2* and are essential for ADP dissociation from DnaA (Kasho et al., 2014).

### Mechanism

Unlike *oriC* or *datA*, DARS sites preferentially bind ADP-DnaA rather than ATP-DnaA. Notably, head-to-head (but not head-to-tail) interactions would occur between the box I-bound DnaA and the box II-bound DnaA, which is a prominent feature at DARS sites (**Figures 1B, 6B**). Formation of oligomers of four or more ADP-DnaA molecules on the *DARS2* core region depends on DnaA AID-2 Leu290, and results in dissociation of ADP (**Figure 6B**). Formation of similar oligomers on the *DARS1* core region promotes dissociation of ADP and is dependent on DnaA AAA+ sensor 1 Asp269, which would participate in probable heat-dependent conformational change of the DnaA nucleotide-binding pocket (Fujimitsu et al., 2009). DnaA sensor 1 Asp269 is dispensable for ADP dissociation at *DARS2* (Kasho et al., 2014), possibly because of still unknown functions of the activator proteins IHF and Fis (**Figure 6B**). Dissociation of ADP produces apo-DnaA molecules that are unstable in binding to the DARS core region, enabling ATP binding and the production of ATP-DnaA (Fujimitsu et al., 2009; Kasho et al., 2014).

Binding of IHF is essential for *DARS2* activity, and, as with DDAH, IHF cannot be replaced by HU. The complex of *DARS2*–IHF–Fis promotes ∼10 times as much ADP dissociation as *DARS1 in vitro* (Kasho et al., 2014). The results of mutational analyses indicate that the spacing between the core-region DnaA boxes, IBS, and FBS is optimized for efficient activation of *DARS2* (Kasho et al., 2014). The sequences and lengths of the spaces between the *DARS2* core region, IBS, and FBS are highly conserved in *E. coli*-proximal β- or γ-proteobacterial species in which IHF and Fis homologs are conserved, suggesting that the regulatory mechanism for *DARS2* is shared among proteobacterial species, including pathogenic species (Kasho et al., 2014).

### Regulation

#### Cell-Cycle-Dependent IHF Binding

IHF binding to *DARS2* is dynamically regulated in a cell-cycle-coordinated manner (Kasho et al., 2014). In contrast to *datA*–IHF binding, *DARS2*–IHF binding occurs specifically in the pre-initiation period, consistent with its role in timely production of ATP-DnaA for replication initiation (Kasho et al., 2014). Regulation of *DARS2*–IHF binding and dissociation is resistant to rifampicin (Kasho et al., 2014), suggesting that it is independent of transcription. The timing of *DARS2*–IHF binding is also independent of replication initiation at *oriC* or replication fork progression (Kasho et al., 2014), supporting the suggestion that functional activation of *DARS2* might be controlled by specific cell cycle (but not DNA replication)-coupled events.

#### Growth-Phase-Dependent Fis Binding

In addition to cell-cycle regulation, growth-phase-dependent regulation contributes to timely activation of *DARS2*. The cellular level of Fis increases specifically in the exponential-growth phase (Ali Azam et al., 1999; Dillon and Dorman, 2010; **Figure 5B**). Fis binding to *DARS2* occurs in the exponential phase, but not in the stationary phase (Kasho et al., 2014). The activation of *DARS2* only in exponential phase is consistent with the requirement for ATP-DnaA for replication initiation.

#### Chromosomal-Position Effect

*Escherichia coli DARS1* (101 bp) and *DARS2* (455 bp) are both located in intergenic regions, at 17.5 min (0.81 Mb position) and 64.0 min (2.97 Mb position), respectively, between *oriC* and *terC* (**Figure 1A**), and these positions are well conserved among related bacteria (Fujimitsu et al., 2009; Kasho et al., 2014; Frimodt-Møller et al., 2015). Translocation of *DARS2* to a *terC*-proximal locus moderately inhibits the timing of initiation, as does its translocation to an *oriC*-proximal locus (Frimodt-Møller et al., 2016; Inoue et al., 2016). Inhibition at the *terC*-proximal locus is sustained in cells lacking MatP, a DNA-binding protein involved in formation of *terC*-specific subchromosomal structure (Mercier et al., 2008; Inoue et al., 2016). The results of 3C (chromosome-conformation-capture) analysis suggest that *oriC* can physically interact with the wild-type *DARS2* locus (Cagliero et al., 2013). These results suggest that the chromosomal location of *DARS2* might be important for efficient interaction with *oriC*. In addition, a specific local chromosomal structure might be important for DARS function.

DnaA activation by *DARS1* and *DARS2* stimulates efficient cell growth under nutrient-poor conditions, and colonization of the large intestine of streptomycin-treated mice (Frimodt-Møller et al., 2015), suggesting a requirement for *DARS1* and *DARS2* for environmental adaptation of *E. coli*. Stimulation of replication initiation by oversupply of *DARS2* has more severe effects on viability under aerobic conditions than under anaerobic conditions (Charbon et al., 2014), suggesting that over-initiation in aerobic conditions leads to lethal levels of encounters between replication forks and sites of oxidative damage (Charbon et al., 2017).

## Perspectives for Coupling Between DnaA Regulation and Cell Growth in *E. coli*

During the *E. coli* cell cycle, levels of ATP-DnaA rise to a peak that induces replication initiation, after which they fall, predominantly as a result of RIDA, a replication-coupled negative-feedback mechanism (Katayama et al., 2010). In the pre-initiation period, *DARS2* is predominant in the conversion of ADP-DnaA to ATP-DnaA. IHF binding to *DARS2* promotes this activity in a timely manner, but IHF is abundant throughout the cell cycle, and the mechanism responsible for its timely binding and dissociation is not yet known. Similarly, the mechanism responsible for the regulation of *datA*–IHF binding, which occurs at a specific time after replication initiation, has not yet been determined. Further studies are required to determine which mechanisms contribute to coupling between these processes and cell-cycle progression. In addition, further studies are required to understand DnaA complex formation/dissociation, ADP dissociation, and ATP hydrolysis during DDAH and in the formation of protein complexes at DARS sites.

Chromosomal positioning of *DARS1, DARS2*, and *datA* is important for regulation of the timing of initiation under specific growth conditions. Chromosomal structural determinants such as superhelicity, co-localization of specific loci in the 3D chromosomal structure, or the timing of changes in copy number during replication that is related to the distance from *oriC* might all contribute to the importance of chromosomal location. Further studies can help to determine which of these mechanisms is relevant for the correct timing of replication initiation.

How DnaB helicase binding to *oriC*-DnaA complexes *in vivo* is regulated is an open question. Even in the presence of a high ATP-DnaA level and *oriC* DUE unwinding, DnaB helicase might still not be able to be bind to *oriC*-DnaA complexes because of the stable interaction between DiaA and DnaA. Further studies of the regulatory mechanisms governing the DiaA binding/dissociation to/from DnaA and DnaB helicase loading *in vivo* are required to understand fully how initiation is timed correctly during the cell cycle.

Intensive research over the last 40 years has increased knowledge of the factors and regulatory mechanisms involved in chromosome replication initiation in *E. coli.* Our knowledge has exploded, and it is possible today to provide a considerably detailed outline about the factors involved and how replication initiation is controlled. At the same time, such progress creates further important mysteries, too. Further work in this area will undoubtedly continue to surprise and inform us about how replication is controlled in this erstwhile considered simple organism and advance knowledge about how this process is controlled in other organisms as well.

## Author Contributions

All authors listed have made a substantial, direct and intellectual contribution to the work, and approved it for publication.

## Conflict of Interest Statement

The authors declare that the research was conducted in the absence of any commercial or financial relationships that could be construed as a potential conflict of interest.
